# Emerging data inputs for infectious diseases surveillance and decision making

**DOI:** 10.3389/fdgth.2023.1131731

**Published:** 2023-04-04

**Authors:** Aminath Shausan, Yoni Nazarathy, Amalie Dyda

**Affiliations:** ^1^ School of Public Health, The University of Queensland, Brisbane, QLD, Australia; ^2^ School of Mathematics and Physics, The University of Queensland, Brisbane, QLD, Australia

**Keywords:** infectious diseases, digital surveillance, emerging data inputs, field experiments, artificial intelligence, crowd sourcing, physiological measures, COVID-19

## Abstract

Infectious diseases create a significant health and social burden globally and can lead to outbreaks and epidemics. Timely surveillance for infectious diseases is required to inform both short and long term public responses and health policies. Novel data inputs for infectious disease surveillance and public health decision making are emerging, accelerated by the COVID-19 pandemic. These include the use of technology-enabled physiological measurements, crowd sourcing, field experiments, and artificial intelligence (AI). These technologies may provide benefits in relation to improved timeliness and reduced resource requirements in comparison to traditional methods. In this review paper, we describe current and emerging data inputs being used for infectious disease surveillance and summarize key benefits and limitations.

## Introduction

Infectious diseases create significant health and social burden globally and can lead to outbreaks and epidemics, highlighted by COVID-19. As of November 29, 2022, there have been almost 638 million confirmed cases and over 6 million deaths of COVID-19 globally.^1^ Prior to this, many other infectious diseases contributed significantly to global mortality and morbidity. Infectious disease threats have arisen in the form of the 2009 Swine Flu pandemic, Middle East respiratory syndrome coronavirus and the West Africa Ebola virus epidemic in West Africa. Cases of newly emerging infectious disease are likely to rise in coming years due to changes in population demographics, climate change, increases in international travel and changes in agriculture, land use, and sanitation (1).

Timely surveillance of infectious diseases is essential to plan targeted long and short term public health response and prevention efforts. Traditionally, infectious disease data are gathered by public health authorities in the form of positive laboratory tests and hospitalization and death data. Various non-government organizations, such as the World Health Organization (WHO) collect and report these data on a global level.^1^ Additional global reporting for COVID-19 specifically has also been implemented by a number of organizations,^2^ (2). However, such data requires significant effort from public health officials and may not be consistently collected on a national or regional scale. There can also be delays in reporting and inconsistencies in reporting of deaths and positive cases. Hospitalization and deaths capture primarily severe cases and may not identify those who have mild symptoms or are asymptomatic. Due to these limitations, these data may not provide the true incidence of the disease.

Over recent years, novel data inputs have emerged to complement traditional surveillance of infectious diseases. This includes the use of sensors and mobile apps to collect symptoms and disease data, crowd sourcing data using Internet based surveys and mobile apps, field experiment data based on scenario analysis, as well as social media and web search data in conjunction with artificial intelligence methods to predict disease outbreaks. Surveillance using these types of data inputs may provide benefits over traditional data sources in that they can provide additional timeliness and are often less resource intensive. We aim to provide an overview of different types of novel data inputs for infectious diseases.

### Online physiological measures

During the early phase of the pandemic, many governments implemented temperature checks, using classical thermometers, as a surveillance method at specific locations. However, this was limited by a single time point for each individual and may not identify all positive cases (3). Real-time temperature reading tools such as the smart thermometer and wearable devices have been tested as potential tools for tracking COVID-19. These tools measure physiological matrices of an individual to identify deviations from a baseline level, which may indicate onset of an illness.

Prior to COVID-19, the smart thermometer has been popularly applied in the US for monitoring flu (Influenza) (4) and personal health risk indicators. During early 2020, data from these thermometers was utilized to forecast COVID-19 hot spots in various US states by correlating anomalously high influenza levels with confirmed COVID-19 cases (5). Similarly, continuous wearable devices were originally applied to track influenza but have been translated to detect COVID-19 (6). Data from smartwatches and Fitbit trackers has been applied to detect pre-symptomatic cases of COVID-19 (7). The WHOOP fitness tracker has been used to identify pre-symptomatic COVID-19 cases by analyzing changes in respiratory rate and heart rate data gathered from users (8).

Data inputs from wearables combined with additional sources have also shown value in surveillance. Prior to the pandemic, the TempTraq skin patch,^3^ a disposable skin patch with wireless temperature monitoring, was used for diagnosis and early detection of health risks. Data from the skin patch with data from Fitbit smartwatches and bio specimens have been used to assist health care workers to self-monitor COVID-19 (9). The TemPredict (10) study investigated whether physiological data such as temperature and heart rate collected by Oura Ring, with self-reported daily symptoms, could predict COVID-19 symptoms. Results showed a correlation between elevated temperature and self-reported fever (11) and algorithms have been developed to identify onset of (12) and recovery from COVID-19 (13). The Corona Data Donation project uses data from wearable devices^4^ with data, such as age and postcode, to provide a daily interactive fever map for COVID-19 hotspots (14). The Digital Engagement and Tracking for Early Control and Treatment (DETECT) study^5^ involved collection of data from volunteers across the US via a smartwatch or activity tracker with self-reported symptoms and diagnostic test results. By evaluating changes in heart rate, sleep, activity levels, and symptoms, these data can identify cases with greater success than symptoms alone (15).

### Crowd sourcing-surveillance

Crowd sourcing involves the collection of information from a large group of people. With the increasing use of the internet these methods are now utilizing digital platforms. For infectious disease surveillance, studies often capture data on self-reported infection via apps or online surveys.

Online surveys for surveillance began in 2003 with the Great Influenza Survey (de Grote Griepmeting) which collects influenza symptoms from participants on a weekly basis. This shows similarity between seasonal influenza measured in the study compared to data reported by general practitioners (16). Tracking influenza through online surveys expanded quickly, with influenza systems now set up in the US (17), Portugal (18), Europe (16), and Australia (19). Since COVID-19 emerged, many of these systems have been adapted to include surveillance for COVID-19 (20,21). The CoronaSurveys (22) and the Global COVID-19 Trends and Impact Survey (23) provide global and country specific daily incidence rates and risk areas for COVID-19. Both surveys are available in multiple languages, attracting participants globally. These types of data collection have shown to provide more timely outbreak signals than testing, hospitalization, and death counts (24). Some systems also incorporate an information feedback loop. The Flu Near You (17) surveillance program tracks influenza symptoms in the US and Canada. Users are able to compare these data with influenza data from the Centers for Disease Control and Prevention sentinel influenza network, and Google Flu Trends. Participants can also identify local sources for influenza vaccination (17).

Numerous mobile phone apps have also been developed to track COVID-19. The COVID Symptom Study app records user reported symptoms.^6^ This data has been used to track population incidence of COVID-19 and to identify hot spots and regions at high risk for COVID-19 (25). The data has been also applied to reveal long-lasting and short-term COVID-19 symptoms (26).

Other studies have focused on monitoring contact patterns and evaluating adherence to social distancing policies. A UK contact tracing survey documented participants age, contact location and level of physical contact (27). Comparing contact patterns during lockdown and social contacts made during a non-epidemic period showed physical distancing measures substantially reduced contact levels. Citizens in China are required to scan a QR code when accessing public spaces to verify their infection status. The QR code provides individual infection risk, based on locations and medical information(28). Contact tracing via QR coding system has been implemented in various other locations, such as the UK (29) and Australia (30).

Crowd sourcing methods are expanding to collect data for a wider range of diseases. The Mo-Buzz system identifies dengue hot spots in the Colombo, Sri Lanka (31). Participants provide dengue symptoms and post pictures of potential dengue mosquito–breeding sites. The system provides data as visual maps and also recommends educational materials. In Puerto Rico, a similar surveillance program called SaludBoricua monitors a number of acute illnesses including influenza, dengue, and leptospirosis (32).

### Novel field experiments

Asymptomatic individuals can also contribute significant risk to the spread of diseases. As discussed previously, various efforts have been made to identify asymptotic individuals via contact tracing. A number of field experiments have also been performed to automatically monitor contact patterns. Data inputs are gathered using applications designed for cellular devices utilizing GPS location tracking.

The UK FluPhone study (33) investigated how social encounters shape the spread of infectious diseases. Participants reported influenza-like symptoms through the Fluphone app. The app also collects proximity of participants’ devices through Bluetooth and location via GPS. Participants were informed about an estimate of the number of encounters they made and a real-time visualization of the spread of virtual SEIR type diseases in the contact network (34). The Safe Blues experiment^7^ is a similar study, which took place at the University of Auckland during 2021–2022 (35,36), examining how physical interactions affect the spread of diseases. The Safe Blues Android app spreads multiple safe virtual virus strands via Bluetooth based on individual’s physical proximity, with strands mimicking the behavior of biological viruses. Data is presented as a real-time and historic dashboard.

The 2018–2019 “Contagion! The BBC Four Pandemic” experiment (37) recruited nearly 30,000 volunteers to download the “BBC Pandemic app.” The app records GPS location during daily activities, and self-reported contacts. Analysis showed that the contact network structure constructed had the potential for understanding and controlling real-world diseases such as the spread of influenza-like viruses (38). The data was also used to simulate various non-pharmaceutical intervention strategies, such as case isolation and social distancing to investigate their effectiveness on limiting the spread of the disease (39).

A number of these experiments have been conducted in the context of mass-gatherings. The RESTART-19 experiment conducted in Germany examined the transmission risks of SARS-CoV-2 in indoor mass-gathering events (40). Using contact tracing devices, participant movements were measured during a concert. This data was incorporated with a computational model of the arena simulating infectious aerosol distribution allowing the development of an individual-based model to estimate the excess burden of the COVID-19 epidemic caused by this event. The study concluded that with moderate hygiene practices and conditions of good ventilation, mass-gathering events contribute little to the spread of COVID-19.

A similar experiment in South Korea recruited healthcare workers involved in two protests (41) to self-report COVID-19 symptoms via an app. Following the rallies, PCR tests were conducted for 609 of the 646 (94%) of the attendees, with all tests negative. However, the PCR tests were performed on a sub sample of attendees and may not provide the true incidence rate. An indoor mass-gathering randomized clinical trial in Spain also assessed the risk of COVID-19 transmission during a concert (42). Contact tracing during the event was performed through a mobile phone app. After eight days, PCR tests showed that less than 1% of participants in the control group had a positive test result and no participant the intervention group tested positive. This provided preliminary evidence on the effectiveness of comprehensive measures to create safe indoor mass-gathering events.

To develop safe practices for mass-gatherings a series of trials called Fieldlab Events were carried out in The Netherlands during February and March 2021.^8^ Four types of experiments investigated the risk of relaxing mask wearing and social distancing measures during mass-gathering events. Contact tracing devices and cameras were used to monitor participants’ behavior during the events. Seven preventive measures were recommended for gatherings in addition to venues adhering to ventilation regulations for indoor events.

### Artificial intelligence assisted data sourcing

Combining artificial intelligence (AI) and information available from informal sources is another surveillance method being applied to infectious diseases. These methods allow analysis and interpretation of large datasets sourced from various sources such as online news and media, expert blogs, validated official alerts (e.g., WHO), clinical reports, social media, and airline data. The most common types of AI methods applied include knowledge-based (KB) techniques, natural language processing (NLP), and machine learning (ML). KB techniques use specific keywords and phrases determined by experts to quickly search through various types of information sources. NLP enables machines to process and interpret human language and perform tasks such as translation and disease classification. ML is the process of applying algorithms that allow machines to automatically learn and improve from experience (43). Table 1 presents key global systems that incorporate AI methods for public health surveillance and their data sources.

**Table 1:** Global surveillance systems incorporating artificial intelligence.

System	Type(s) of surveillance	AI methods	Data sources
GPHIN	Disease outbreaks, infectious diseases, contaminated food and water bioterrorism and exposure to chemicals, natural disasters, and issues related to the safety of products, drugs and medical devices and radioactive agents	KB, ML, NLP	Newspapers, online articles, emails, expert blogs, public health bodies and social media
HealthMap	Infectious disease outbreaks	ML, NLP	News media (e.g., Google News), expert-curated accounts (e.g., ProMED Mail), and validated official alerts World Health Organization announcements
BlueDot	Infectious disease outbreaks and risk assessments	ML, NLP	Online articles and airline data
Metabiota	Infectious disease outbreaks and risk assessments	ML, NLP	Data from various communities and regional clinics, online reports, social media data and airline data
WHO-EIOS	Outbreaks, risk assessments, pattern discovery and forecasting new outbreaks of all diseases	ML, NLP	Online media and specific social media sources, government and official web sites, news aggregators, blogs and expert groups, and collaborating initiatives (ProMED, GPHIN, HealthMap and the Europe Media Monitors
BioCaster	Infectious disease outbreaks	ML, NLP	Google News, ProMed Mail, MedISys, WHO, The United Nations, The European Centre for Disease Prevention and Control, Center for Infectious Disease Research and Policy, EurekAlert!, China News Service, The Food and Agriculture Organization of the United Nations
MedISys	Monitoring infectious diseases, bioterrorism, and chemical, biological, radiological and nuclear threats	KB, NLP, ML	Online articles

The Global Public Health Intelligence Network (GPHIN^9^) was the first digital tool providing early warnings for diseases worldwide (44), and provided early detection of SARS in 2002 (45). The system automatically extracts data from media aggregators such as Google News, other online news, public health organizations and expert blogs, which are analyzed using KB, NLP, and ML methods. These findings are validated and fine tuned by experts and displayed as maps, graphs and listed documents (45). Initially the system monitored human diseases, however the platform has been expanded to include food safety surveillance.

The HealthMap,^10^ BlueDot^11^ and Metabiota^12^ platforms integrate data from a range of formal and informal sources including news media such as Google News, expert curated accounts such as ProMed-Mail, social media and validated official alerts such as WHO (46). These systems use NLP and ML to analyse these data for public health use. HealthMap was among the first systems to issue an early warning of COVID-19 outbreak 11 days prior to WHO confirming the disease (46). BlueDot (47) reported COVID-19 outbreaks 10 days before the disease was confirmed by WHO, and has successfully identified urban areas with high risk of spreading the disease (48).

The Epidemic Intelligence from Open Sources (EIOS^13^) is an initiative of WHO which integrates existing and new surveillance initiatives, networks, and systems globally (49). Data is gathered from a range of sources including traditional online media, social media, government and official web sites, blogs, and collaborating initiatives, such as the HealthMap and ProMed-Mail. Using NLP and ML the system sorts and categorizes articles by topics, country, language, source and contextual indices. Data from both EIOS and BlueDot were used to enhance event-based surveillance in Japan during the 2020 Olympic and Paralympic Summer Games, which helped to identify events, characterize risk and reduced the surveillance workload of Japan (50).

BioCaster^14^ analyzes data from local and international news, sourced from news aggregators such as Google News and RSS feeds. Using NLP and ML the system automatically provides information of disease prevalence and outbreaks specific to a given location, as visual maps and graphs (51). Similarly, the Medical Information System (MedISys^15^) monitors events occurring across the globe using information from news articles (52). The system curates data from official government websites, health news and the general media, and performs categorization of a range of public health threats by applying KB, ML and NLP techniques.

## Discussion

These data inputs have several strengths in comparison to traditional methods. A key strength is timeliness as they often rely on automatic data collection. They may also reach to a wider population, more localized areas and population groups, and thus data collected from these systems can be more comprehensive. They can capture important aspects of a disease or transmission, such as changing behavior and perceptions regarding public health policies. These systems also require less human capital to run, and hence are less costly. When all aspects of digital systems are integrated, including data collection, analysis and presentation, this increases flexibility. Despite these benefits, there are notable limitations.

Tools measuring physiological indicators have limited specificity as they often rely on syndromic definitions of diseases, and there is difficulty in adjusting for confounders. Gathering more information in relation to demographics and risk factors to address this may lead to decreased in participation (53). Some systems using physiological measurements, as well as crowd sourcing, phone data, and mobile apps depend on user generated data. This method of data collection has inherent challenges, including introducing bias as those who choose to participate may not be representative of the general population. Data may also be biased due to user demography. Crowd sourcing can also require a participation over a long period time with recruitment of participants and ongoing engagement challenging.

Similarly, field experiments require a large number of participants making such experiments challenging to implement. This is highlighted by the lengthy disruption in the Safe Blues experiment due to the lockdown started during August 2021 in New Zealand (36). Furthermore, the results of these studies are restricted to specific populations, and may not be useful for different population groups or locations.

While powerful, mobile phone apps based on contact tracing, QR codes, and GPS based location trackers can create privacy and ethical challenges (54), and thus can be inefficient due to low user uptake (55,56). Privacy and ethical concerns can also arise from social media and web search data, due to the fact that such data are not primarily health related, often include personal characteristics, and owned by private online platforms (57). Such data can incorrectly predict prevalence due to having irrelevant information in search outputs or changing user behavior (58).

AI enabled systems collect and interpret data from a broad range of sources in real time and can potentially monitor disease trends and detect emerging diseases. However, this technology requires a large volume of data to make useful insights, and specialist computational infrastructure is needed to manage and store such data. Additionally, many of these data sources are unstructured and contain missing information and errors which again requires specific skills to analyze. As many of the AI enabled system integrate data from social media, they may not be representative of the population, with younger people over-represented in the samples (59).

In summary, new digital technologies provide benefits and opportunities to improve disease surveillance globally. However, the limitations of these data inputs and associated methods must be understood and addressed when incorporated into public health practice.

## Footnotes

^1^ https://covid19.who.int

^2^ www.ncov2019.live

^3^ www.temptraq.healthcare

^4^ www.corona-datenspende.de/science/en

^5^ https://detect.scripps.edu

^6^ https://health-study.joinzoe.com/us-2

^7^ www.safeblues.org

^8^ www.fieldlabevenementen.nl/fieldlab-english

^9^ www.gphin.canada.ca

^10^ www.healthmap.org

^11^ www.bluedot.global

^12^ www.metabiota.com

^13^ www.who.int/initiatives/eios

^14^ www.biocaster.org

^15^ MedISys.newsbrief.eu
